# Case Report: Novel compound heterozygous mutations in *SLC34A2* gene: a case of pulmonary alveolar microlithiasis in a child

**DOI:** 10.3389/fped.2026.1646732

**Published:** 2026-03-09

**Authors:** Tong Zhou, Shangge Xu, Jinghua Yang, Yiting Chen

**Affiliations:** 1 Department of Pediatrics, The Second Affiliated Hospital, Guangzhou University of Chinese Medicine, Guangzhou, China; 2 The Second Clinical Medical College, Guangzhou University of Chinese Medicine, Guangzhou, China

**Keywords:** children, compound heterozygous mutations, missed diagnosis, pulmonary alveolar microlithiasis, *SLC34A2*

## Abstract

Pulmonary alveolar microlithiasis (PAM), a rare disorder caused by *SLC34A2* mutations, demonstrates clinical-radiological discordance. Pediatric cases face heightened diagnostic challenges; nonspecific presentations often result in misattribution to recurrent pneumonia or delayed diagnosis, especially in children ≤5 years. We present a case of a 3-year-old girl hospitalized for persistent cough (11-day duration) and intermittent fever (9-day history). Her recurrent productive cough had been misattributed to recurrent pneumonia in prior healthcare encounters. Diagnostic imaging revealed extensive calcifications in the left lower lobe on chest CT, accompanied by small clustered onion-like calcifications in bronchoalveolar lavage fluid(BALF). Sputum culture identified *Haemophilus influenzae* infection. Genetic analysis revealed novel compound heterozygous variants in *SLC34A2*: c.524-1G>C (IVS5) inherited maternally and c.910A>T (EX8) of paternal origin. The patient was diagnosed with PAM complicated by *Haemophilus influenzae* pneumonia. These compound heterozygous *SLC34A2* variants represent previously unreported pathogenic mutations. We conclude that heightened attention should be directed toward detecting calcifications on mediastinal and bone windows during pediatric imaging examinations. Genetic analysis plays a pivotal role in diagnosing rare childhood disorders and may emerge as the primary diagnostic modality for PAM in pediatric populations.

## 1 Introduction

Pulmonary alveolar microlithiasis (PAM) is a rare autosomal recessive disorder caused by mutations in *SLC34A2*, which encodes the type IIb sodium-dependent phosphate cotransporter (Npt2b) (1). Pathogenic variants in this gene result in defective phosphate transport, leading to diffuse calcium-phosphate microlith deposition within alveolar spaces and pulmonary interstitium. Clinically, PAM progresses chronically without associated calcium-phosphorus metabolic abnormalities, ultimately causing respiratory failure, pulmonary hypertension, and fibrosis (2, 3). The disease demonstrates a high familial incidence (38%–61%) and manifests across all age groups, from neonates to octogenarians (4–6). Most patients are asymptomatic, and the disease is discovered incidentally. Characterized by the clinical-radiological dissociation, diagnosis bases on hallmark imaging features or genetic confirmation. Current therapeutic options remain limited, with lung transplantation representing the only definitive intervention.

## 2 Case presentation

On May 22, 2024, a 3-year-old, 11-month-old girl was admitted to our hospital with an 11-day cough and 9-day intermittent fever. Lab results showed leukocytosis, neutrophilia, elevated hs-CRP, and the antibody to *Mycoplasma pneumoniae* was positive (1:640). chest radiography revealed left lower lobe-predominant bilateral infiltrates. The child was treated with cefaclor for 3 days and azithromycin for 1 day in the outpatient clinic, but the results were unsatisfactory, and then she was admitted to the hospital.

Upon admission, the girl presented with a body temperature of 37.6 °C, respiratory rate of 24 breaths/min, heart rate of 135 beats/min, blood pressure of 92/56 mmHg, and oxygen saturation of 96%. Physical examination revealed mild intercostal retractions, slightly coarse breath sounds in both lungs, and fine crackles in the left lung. Laboratory findings demonstrated elevated immunological markers with total IgE of 648.2 IU/mL, IgA of 2.42 g/L, and IgM of 2.26 g/L. The results of the complete blood count and inflammatory markers are presented in Table 1. Biochemical profile (including liver function tests, cardiac biomarkers, renal profile, and lipid panel), ASO titers, autoimmune antibody panel (12-item), and anticardiolipin antibodies showed no abnormalities. Arterial blood gas analysis under room air (FiO_2_ 21%) revealed pH 7.441, PO_2_ 83.5 mmHg, and PCO₂ 32.0 mmHg. Sputum culture with Gram staining confirmed *Haemophilus influenzae(H. influenzae)* infection demonstrating azithromycin non-susceptibility.

**Table 1:** The findings of laboratory test.

Date/Day of illness	2024/5/14 (D3)	2024/5/21 (D10)	2024/5/22 (D11)	2024/5/23(D12)	2024/5/31 (D20)
WBCx10^9/L (4.4–11.9)	13.42	16.55	12,74		8.57
NEUTx10^9/L (1.2–7.0)	11,13	11.42	8.31		3.21
NEUT% (22.0–65.0)	82.9	69	65.3		37.4
LYMx10^9/L (1.8–6.3)	1.34	3.59	3.06		4.36
LYM% (23.0–69.0)	10.0	21.7	24.0		50.9
RBCx10^12/L (4.0–5.5)	4.44	4.18	4.07		4.57
Hb,g/L (112–149)	120	116	105		118
PLTx10^9/L (188–472)	272	315	387		501
hsCRP,mg/L (0.00–6.00)	8.13	42.93	23.43		3.05
ESR (0–32.0)			85		
Ferritin,ng/mL (4.63–204.0)				171.45	
IL-6 (0.00–7.00)			10.45		
total IgE (0–60)				648.2	
PCT (0–0.05)			0.29		

The patient was an only child, first born at term, delivered by caesarean section due to malposition, with a birth weight of 3 kg. His parents were from the same village in Hunan province, and were not consanguineous, with no family history of similar diseases. She resided in Hunan until age 1 near an operational coal mining facility, subsequently relocating to Guangzhou. There was no history of asthma or other allergic diseases. She had a normal nutritional status (height on 28.1st percentile for weight on 6.1st percentile) and normal breathing pattern.

**Figure 1:**
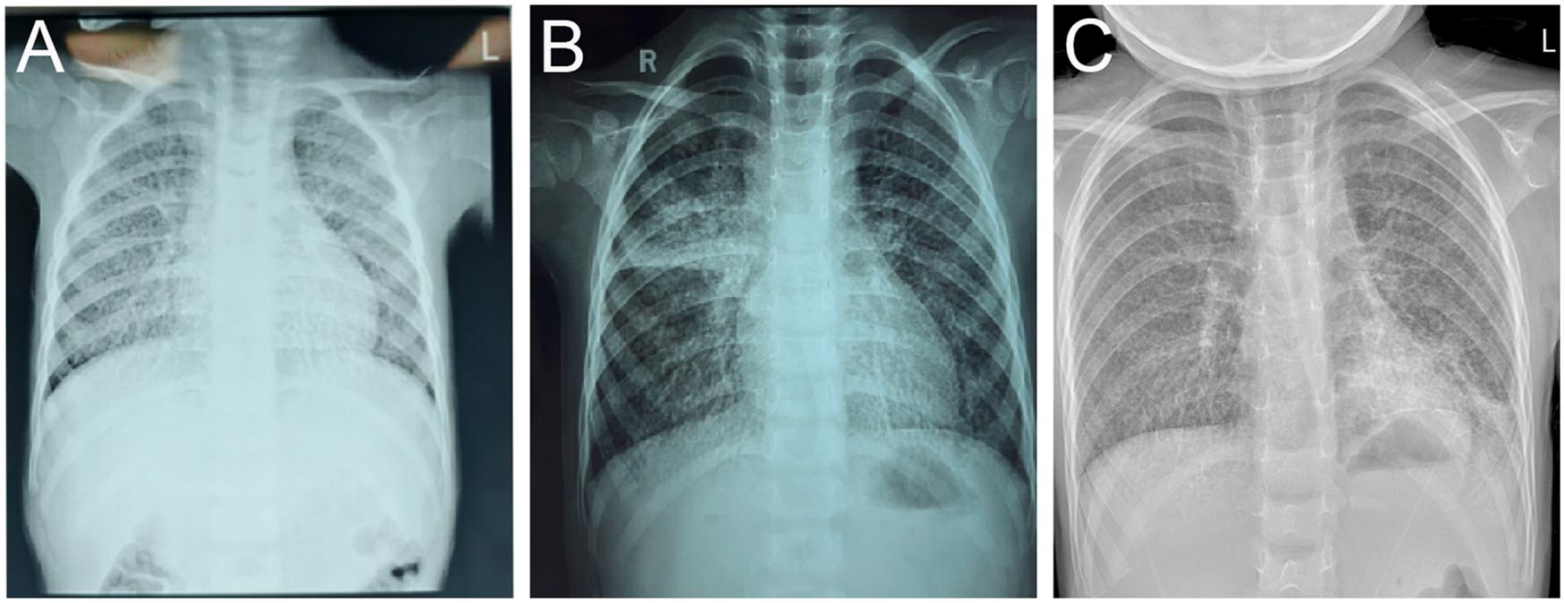
**(A)** August 2023. The patient presented to an external hospital with fever, cough, and rhinorrhea. Chest radiography revealed increased opacity in both lung fields with small patchy opacities distributed along bronchial patterns, suggestive of bronchopneumonia. The outpatient physician administered azithromycin followed by cefuroxime, leading to symptomatic improvement, but the condition was prone to recurrence. **(B)** October 2023. The patient presented to another hospital with fever and cough. Chest radiography revealed diffuse interstitial changes with localized consolidation in both lungs, clinically diagnosed as bronchopneumonia. The outpatient physician administered intravenous ceftriaxone sodium and oral azithromycin for infection control, resulting in symptomatic improvement but incomplete resolution. **(C)** Chest radiography obtained upon current admission.

A chest radiography demonstrated bilateral interstitial abnormalities. Further history-taking revealed recurrent cough episodes over the past year, including two confirmed pneumonia diagnoses. We were able to obtain chest radiographs from these two prior episodes of pneumonia. However, as the child was treated empirically in outpatient settings at external institutions at the time, no further detailed laboratory testing was performed. Therefore, our interpretation of the likely infectious etiology is necessarily based on the documented clinical presentation and management provided during those visits.Although previous symptoms showed responsiveness to therapeutic interventions, historical chest radiography consistently demonstrated interstitial abnormalities (Figure 1).

**Figure 2:**
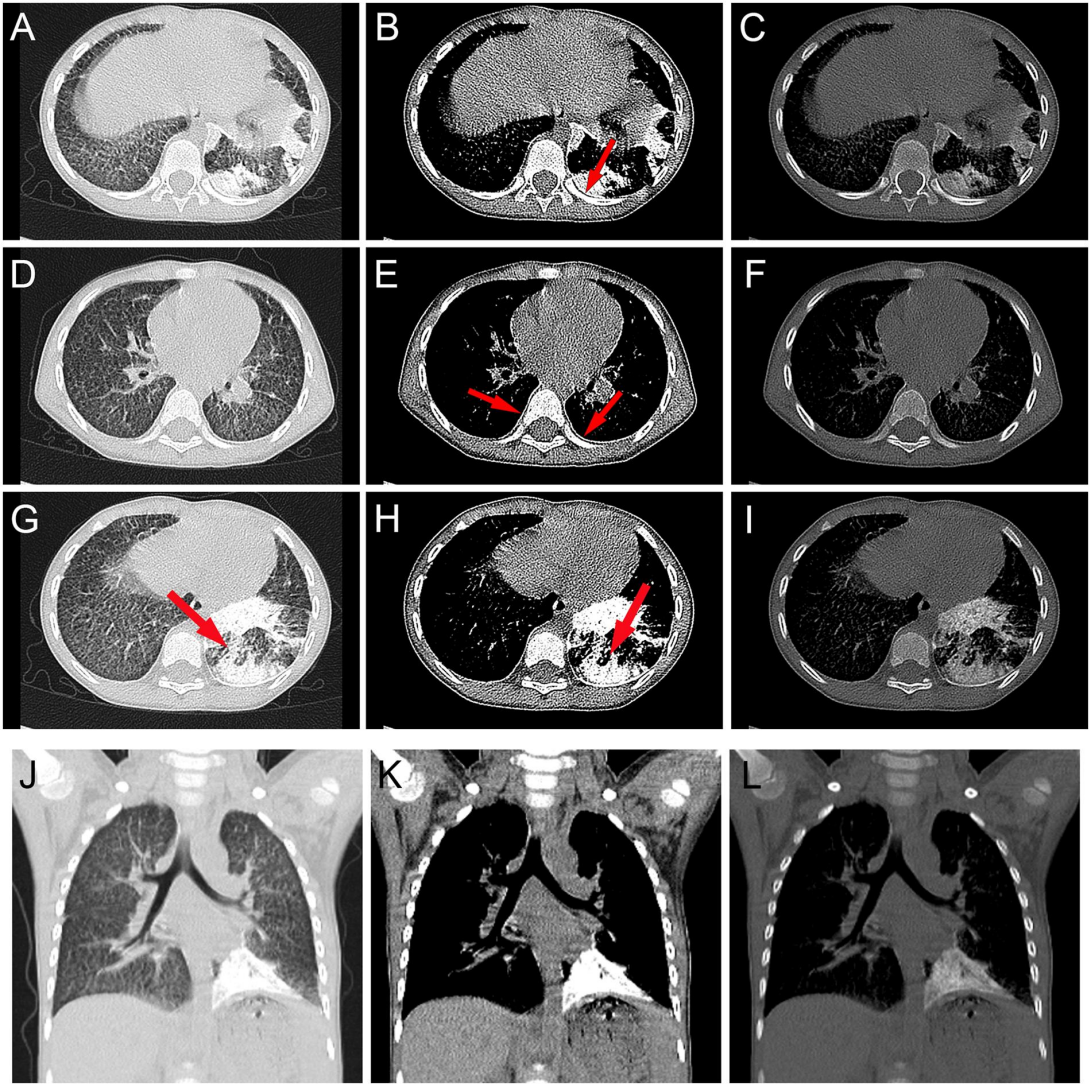
The HRCT of the patient demonstrates: **(A,D,G,J)** lung window, **(B,E,H,K)** mediastinal window, and **(C,F,I,L)** bone window views. Calcifications along the cardiac border and pleura were distinctly visualized in both mediastinal and bone windows. **(B)** Subpleural cysts between the ribs and calcified parenchyma were referred to as the “black pleura” sign (red arrow). **(E)** The “white line sign” (red arrow) was characterized by multiple punctate and linear calcifications. **(G,H)** The “flame-shaped sign” **(J,K,L)** Extensive calcifications are observed along the left lower pulmonary-diaphragmatic interface.

Thickening of the interlobular septa in both lungs and extensive calcifications in the left lower lobe were demonstrated on high-resolution computed tomography (HRCT) scan (Figure 2). These calcifications appeared as the typical “black pleural sign” “white line sign” and “flame-shaped sign”. Targeted sequencing of multiple respiratory pathogens in the bronchoalveolar lavage fluid (BALF) identified the presence of *H. influenzae* (homogenized sequence number 45210). *H. influenzae* was also cultured from the alveolar lavage fluid. Testing for *Mycobacterium tuberculosis*(*M. tuberculosis*) and rifampicin resistance, as well as the Purified Protein Derivative(PPD), were negative. Nucleic acid tests for antigens of influenza A and B viruses, *Streptococcus pneumoniae* antigens, *Mycoplasma pneumoniae* DNA, and six respiratory viruses all yielded negative results.

We conducted an etiological search of the pulmonary calcification lesion. The patient underwent comprehensive color Doppler ultrasound examinations of the abdomen, cardiac system, urinary system, and adrenal glands, which revealed no calcifications in other organs. Autoimmune screening (12-item panel), immunological tests (5-item panel), and anti-cardiolipin antibody assays showed no significant abnormalities, thereby excluding interstitial lung disease. Normal parathyroid hormone levels, calcium and phosphorus homeostasis, and 25-hydroxyvitamin D concentrations ruled out parathyroid disorders and calcium-phosphorus metabolic disturbances. Negative results from the PPD test combined with *M. tuberculosis* detection and rifampicin resistance assay excluded pulmonary tuberculosis. Based on negative results from sputum smears, sputum cultures, bronchoalveolar lavage fluid metagenomic next-generation sequencing (BALF mNGS), and serum (1,3)-β-D-glucan testing, along with the absence of characteristic imaging features of invasive fungal infection on chest CT, fungal infection was ruled out.

Regarding the girl's history of special environmental exposure before one year of age, the possibility of pneumoconiosis was considered. However, the typical chest CT findings of pneumoconiosis primarily include nodular opacities, interstitial fibrosis, architectural distortion with emphysema, and pleural changes (7–10). Although calcification may occur in some nodules, they are generally uniformly distributed, of homogeneous density, and well-defined—features inconsistent with the chest CT presentation observed in this child. Furthermore, pneumoconiosis is definitively associated with prolonged, high-dose occupational exposure. It would be highly atypical for such a brief exposure during infancy to result in the characteristic radiological manifestations, making this history inconsistent with the typical profile of pneumoconiosis.

**Figure 3:**
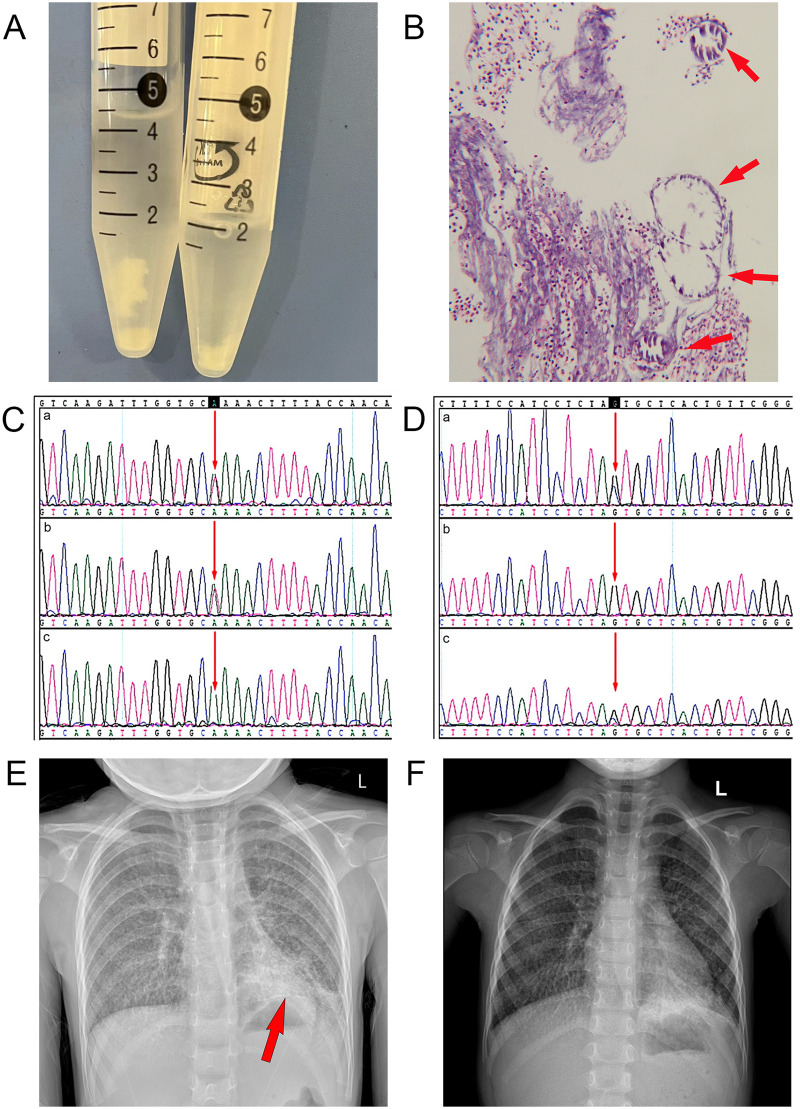
**(A)** macroscopic white sediment was observed in the BALF specimens. **(B)** Pathological examination of BALF revealed abundant neutrophils and small calcified clusters with concentric onion-like patterns. **(C,D)** Genetic analysis of the girl and parents. **(C)** Paternally inherited *SLC34A2* exon 8 mutation c.910A>T (p.Lys304*) **(D)** Maternally inherited *SLC34A2* intron 5 mutation c.524-1G>C **(a)** PAM patient; **(b)** father; **(c)** mother; **(E)** Initial chest radiography demonstrating bilateral pulmonary infiltrates with left lower lobe predominance and a linear calcific opacity (arrow). **(F)** Pre-discharge chest radiography showing persistent bilateral pulmonary infiltrates with partial resolution in the left lower lobe.

In pediatric cases, pulmonary alveolar proteinosis (PAP) can exhibit a “crazy-paving signs” on imaging similar to that seen in PAM. Cytological analysis of BALF serves as one of the most distinguishing diagnostic clues between the two. Typically, BALF in PAP appears milky or turbid with a lipid-rich quality and lacks visible solid sediment (11, 12). In contrast, the BALF from this patient was not milky or turbid; rather, it was clear with visible white particulate sediment. Pathological examination further revealed abundant neutrophils and small calcific clusters under microscopy (Figure 3).Pulmonary function testing could not be performed due to the girl's young age, and lung biopsy was not performed due to parental refusal. Comprehensive whole-exome sequencing of the girl and her parents was conducted for diagnostic clarification. Genetic analysis identified two pathogenic compound heterozygous variants in the *SLC34A2* gene associated with PAM: c.524-1G>C (IVS5/AC4) (maternally inherited) and c.910A>T (EX8/CDS7) (paternally inherited) (Figure 3). Both variants were classified as pathogenic according to the American College of Medical Genetics and Genomics (ACMG) guidelines (13). Based on these findings, the patient was definitively diagnosed with PAM and *H. influenzae* pneumonia.

Empiric azithromycin therapy was administered orally for 4 days during hospitalization, followed by targeted ceftriaxone anti-infective treatment guided by laboratory results. The therapeutic regimen included combined bronchodilators (budesonide and albuterol), mechanical airway clearance, and traditional Chinese medicine. The patient demonstrated clinical improvement and was discharged. Pre-discharge chest radiography revealed persistent bilateral pulmonary infiltrates with partial resolution in the left lower lobe (Figure 3). The child was under continuous outpatient follow-up at our institution until May 2025. Throughout this period, the patient experienced persistent symptoms of recurrent cough and expectoration, for which traditional Chinese medicine was administered in our clinic. Regrettably, after this point, the family voluntarily discontinued follow-up contact. During the final communication, they explicitly declined any further CT or other radiological imaging due to concerns regarding radiation exposure. They also maintained that additional testing would not benefit the child's current condition and expressed a wish to avoid causing further distress to the child.

## 3 Discussion

PAM is characterized by diffuse intra-alveolar sand-like calcifications composed of calcium and phosphate. This disease has been reported worldwide, with higher prevalence in consanguineous populations, particularly in Turkey, Japan, India, and Italy. Both familial and sporadic cases exist, with familial clustering documented in 32%–61% of reported cases (14). Notably, horizontal transmission among siblings and cousins predominates over vertical parent-child inheritance in most familial cases, consistent with an autosomal recessive inheritance pattern (15–17).

The *SLC34A2* gene, identified as the causative gene for PAM, encodes a sodium-dependent phosphate transporter (NaPi-IIb) expressed in type II pneumocytes. Mutations in this gene impair phosphate transport activity, leading to pathological accumulation of phosphate derived from phospholipid metabolism in alveoli. This retained phosphate binds with calcium to form calcium phosphate microliths, which deposit within alveolar spaces (1, 18). Globally, 34 allelic variants have been identified in 68 genetically confirmed PAM cases, predominantly in homozygous states (19). Previously reported compound heterozygous variants typically combine a missense mutation with either a nonsense or splice-site variant (20–24). We identified a novel compound heterozygous mutation: a maternally inherited intronic splice-site variant (c.524-1G>C, IVS5) predicted to cause aberrant mRNA splicing, and a paternally inherited exonic nonsense variant (c.910A>T, p.Lys304*) resulting in premature protein truncation. To date, both variants have only been reported in Chinese populations: the c.524-1G>C splice-site variant was observed in two Chinese PAM cases (23, 25), and the c.910A>T nonsense variant appears to demonstrate elevated recurrence specifically within this ethnic group (22, 26–28). These observations suggest that intron 5 and exon 8 of *SLC34A2* may represent prioritized screening regions for Chinese PAM patients, with the co-occurrence of splice-altering and truncating variants providing preliminary evidence for their potential contribution to disease pathogenesis through complementary loss-of-function mechanisms.

PAM is characterized by clinical-radiological dissociation—patients remain asymptomatic despite exhibiting striking “sandstorm-like” diffuse calcifications on chest radiography. This paradoxical presentation may involve two interrelated mechanisms:

·Preserved Pulmonary Compliance: Early-stage microlith deposition spares alveolar elasticity, enabling silent disease progression despite severe radiographic changes.

· Infection-Mediated Masking: Concurrent infections generate inflammatory infiltrates that obscure diagnostic calcifications on standard CT lung windows.

The disease follows an indolent course, with diagnosis predominantly established in the second to fourth decades of life. Adult manifestations classically include exertional dyspnea, dry cough, chest pain, fatigue, and hemoptysis. Pediatric cases are exceptionally rare, with diagnostic rates particularly low (2%–3%) in children ≤5 years old (14, 29), showing male predominance. Affected children often present with nonspecific symptoms (fever, cough, or acute hypoxemic respiratory failure) that mimic common childhood respiratory infections (29, 30). Our patient developed recurrent cough from age 3, achieving definitive diagnosis within 1 year of symptom onset—a notably accelerated timeline compared to the decade-long diagnostic delays typically seen in adults. Notably, the co-infection with *Haemophilus influenzae* complicated the clinical presentation. A review of the literature on pediatric PAM patients reveals that only a limited number of cases describe co-infections, and there is a lack of discussion on mechanisms underlying susceptibility to specific pathogens. Some researchers have speculated that the pulmonary pathology associated with PAM may increase susceptibility to severe respiratory infections from opportunistic pathogens (31). We propose that PAM, as a structural lung disease, does not directly confer a pathogen-specific susceptibility. Instead, the pulmonary fibrosis and impaired lung function caused by PAM likely compromise the host's defense against respiratory infections. Studies suggest that microliths can induce osteoclast-like differentiation of lung monocytes, potentially leading to a partial or complete loss of their host defense functions (32). Therefore, in this 3-year-old patient, the *H. influenzae* co-infection is more likely attributable to a compromised local defense resulting from the altered pulmonary microenvironment in PAM, rather than to a disease-specific susceptibility. Taken together, the diagnosis of PAM would have been entirely overlooked without meticulous evaluation of mediastinal and bone window calcifications on CT, particularly in the context of confounding co-infections such as *H. influenzae*.

The HRCT of the girl revealed extensive calcifications in the left lower lung field, a finding rarely documented in previous studies, particularly among young children. Compared to all age groups, the ≤5-year cohort demonstrates significantly lower incidence and less pronounced calcification severity. This case represents a notably young pediatric PAM patient with severe radiological manifestations. Notably, imaging findings typically progress with disease advancement, and under standard radiological staging criteria, most pediatric PAM cases are classified as Stage I or II. However, she was classified as Stage III, necessitating urgent therapeutic intervention (14, 33). Concurrent respiratory tract infection may accelerate PAM progression, as reported by previous reports (34). Although nebulized therapy and targeted antibiotics achieved partial clinical improvement, follow-up evaluations revealed persistent cough and recurrent bronchitis episodes, indicating incomplete resolution of symptoms.

Given the rarity of PAM, recognition of characteristic thoracic imaging signs—including “crazy paving signs,” “black pleural sign,” and “white line sign”—remains limited among clinicians. Consequently, definitive diagnosis necessitates pathological confirmation or genetic testing for *SLC34A2* mutations. Early diagnosis is of paramount importance in pediatric patients. Beyond avoiding unnecessary antibiotic use and invasive procedures, it facilitates prompt genetic testing for family members, enabling early screening and preventive counseling (35). Although no curative therapy currently exists to reverse the pathological changes, an early diagnosis allows clinicians to implement supportive measures—such as anti-inflammatory treatment and ventilation support—during the indolent phase of the disease, thereby slowing the rate of pulmonary functional decline. No effective therapies currently exist for PAM, with bisphosphonate treatment remaining controversial. Serial bronchoalveolar lavage and systemic corticosteroids demonstrate no therapeutic efficacy (36–38). Lung transplantation offers definitive management for end-stage disease, with successful cases documented globally (35). No post-transplant recurrence has been reported to date, though current longest post-transplant survival stands at 7.5 years—a limitation reflecting short follow-up durations in most studies (39). Long-term outcomes require further investigation (40–42).No established guidelines govern transplant referral timing for PAM, with no documented cases in children. Some experts recommend referral upon right heart failure or oxygen-dependent respiratory failure (43). Early referral prior to severe right ventricular dysfunction optimizes outcomes. For pediatric PAM, we advocate pulmonary function tests, thoracic CT surveillance, and 6-minute walk tests to determine optimal referral timing. While traditional Chinese medicine (TCM) efficacy remains unproven, our patient demonstrated symptomatic improvement through outpatient TCM interventions.

We recommended preliminary imaging screening for the patient's first-degree relatives. None of the relatives reported relevant clinical symptoms. Chest x-rays were performed, showing no significant abnormalities. In line with radiation protection principles and in the absence of initial positive findings, further CT examinations were not arranged at this time. The importance of regular follow-up was communicated to the family. Additionally, we provided comprehensive genetic counseling to the family regarding future reproductive options, such as prenatal diagnosis or preimplantation genetic testing. After receiving this information, the family indicated they have no immediate plans for another pregnancy but expressed understanding and appreciation for the details provided. They are now aware that further genetic counseling is available should the need arise in the future.

However, this case report has limitations due to the incomplete pulmonary function tests and the lack of comprehensive follow-up CT imaging, which preclude an objective assessment of therapeutic efficacy. Despite these constraints, we believe this report holds significant value. It clearly illustrates the diagnostic challenges in identifying PAM in young children, particularly when symptoms overlap with common respiratory infections. This underscores the importance of maintaining a high index of suspicion for alternative diagnoses in children with “refractory” or “recurrent” pneumonia. During the available follow-up period, we observed the persistence of clinical symptoms in the setting of co-infection, offering a clinical clue to the potential “triggering” or “exacerbating” role infections may play in PAM. Furthermore, the family's concerns regarding investigations and their subsequent loss to follow-up reflect the practical challenges in managing rare chronic pulmonary diseases in children, encompassing issues of long-term treatment adherence and familial risk-benefit considerations.

## 4 Conclusion

We report a genetically and radiologically confirmed case of PAM in a 3-year-old female. *SLC34A2*-associated PAM represents a rare pediatric interstitial lung disease frequently misdiagnosed due to nonspecific symptoms (29). The condition remains exceptionally uncommon in children under five years of age, likely attributable to subtle clinical manifestations and underrecognized imaging features. We emphasize meticulous evaluation of mediastinal and bone windows for pulmonary calcifications in pediatric imaging. Children exhibiting diffuse interstitial abnormalities with extensive calcifications warrant consideration of PAM.

This case was diagnosed through identification of two novel *SLC34A2* variants, expanding the mutational spectrum of PAM. Current evidence demonstrates no established genotype-phenotype correlations, necessitating larger cohort studies. Emerging research suggests potential associations between genetic variant profiles and phenotypic severity in PAM (20). This unique case provides insights into PAM pathogenesis and underscores the diagnostic imperative of genetic analysis in rare pediatric disorders. As of the end of 2025, no published preclinical studies have successfully demonstrated gene correction using Clustered Regularly Interspaced Short Palindromic Repeaths technology in animal models of PAM (19). Gene therapy is considered a promising future direction, as it is expected to achieve long-term or permanent expression in target cells. To lay the groundwork for potential gene-based treatments, we recommend implementing broader genetic testing in patients and systematically evaluating the functional characteristics and distribution patterns of the variant spectrum.

## Data Availability

The original contributions presented in the study are included in the article/Supplementary Material, further inquiries can be directed to the corresponding author/s.
